# Breath of Relief: Osteopathic Manipulative Treatment of Chronic Postoperative Pain in a Bilateral Lung Transplant Patient

**DOI:** 10.7759/cureus.84183

**Published:** 2025-05-15

**Authors:** Vaibhav Duggal, Alexa Finkelstein, Rachel Radigan, Priya Bhushan

**Affiliations:** 1 Osteopathic Manipulative Medicine, New York Institute of Technology, College of Osteopathic Medicine, Old Westbury, USA

**Keywords:** chronic postoperative pain, dermatomyositis, lung transplant, osteopathic manipulative medicine, osteopathy treatment, post-lung transplant complications

## Abstract

Critical stage lung disease often requires treatment with a lung transplant. This procedure can change the quality of a patient’s life, but it does not come without its potential complications. Chronic postoperative pain is one such complication, which is underaddressed and not properly managed. While current management revolves around the use of medications, it is often insufficient. Osteopathic manipulative treatment (OMT) is an additional means of treatment that has been well studied in its ability to address somatic dysfunctions, decrease stiffness, improve respiratory function, and minimize pain. Our 56-year-old patient, who was status-post bilateral lung transplant in 2022, presented to our clinic due to chronic back pain, stiffness, inspiratory pain, and generalized weakness. While her vitals were within normal limits and her general physical exam was benign, her osteopathic structural exam was significant. Alongside thoracic and lumbar restrictions, she presented with hypertonicity of her right latissimus dorsi, serratus anterior, subscapularis, and psoas muscles. She also had articular restrictions of her right ribs. After undergoing OMT for the next five months, the patient continued to report extended bouts of pain relief as well as a decrease in pain with deep inhalation. Her initially reported six out of 10 pain became a consistent two out of 10. She also reported an increase in back strength, a reduction in stiffness, and an overall increase in comfort, allowing her to sleep easily at night. While bringing more attention to chronic postoperative pain, this case demonstrates the efficacy of OMT in becoming an additional part of its management.

## Introduction

Lung transplant is a life-saving treatment for patients whose lung disease has reached a critical stage when they are no longer responsive to medical treatment. Although often considered one of the most difficult procedures to perform and recover from, lung transplants make a major difference, providing improved quality of life [1]. Over 4,500 lung transplants are performed yearly worldwide, with over half being in North America [2]. Careful considerations must be made to determine if a patient is suited to undergo a lung transplant. The general criteria include patients with chronic end-stage lung disease who have a greater than 50% risk of death within two years if the transplant is not performed, a greater than 80% likelihood of surviving at least 90 days after lung transplant, and a greater than 80% likelihood of five-year post-transplant survival. The Lung Allocation Score (LAS) can be utilized to improve outcomes by aiding in the determination of which patients to prioritize. The score consists of 12 factors that influence individual mortality with advanced lung disease. It is determined by subtracting the predicted one-year survival without transplant from the predicted one-year survival with transplant [1]. Patients with higher scores often receive priority in receiving the donor lung. As with any procedure, there are contraindications to attempting a lung transplant. A recent history of malignancy, significant dysfunction of another organ, uncontrollable bleeding disorders, or even non-compliance with medical therapy are all absolute contraindications to lung transplantation.

The disease process resulting in end-stage lung disease necessitating transplant varies between patients. Conditions such as cystic fibrosis, chronic obstructive pulmonary disease (COPD), interstitial lung disease, sarcoidosis, idiopathic pulmonary hypertension, and other autoimmune conditions can all necessitate a lung transplant. Dermatomyositis is a rare acquired autoimmune disorder often characterized by symmetric proximal muscle weakness and skin rash. It is thought to be a result of a humoral-mediated attack against muscle capillaries and the endothelium of arterioles, resulting in decreased capillary density and degeneration of muscle fibers [3]. The development of severe interstitial lung disease, which occurs in one-third of patients, is associated with higher mortality. Management of the lung disease may require transplantation.

When lung transplantation is required, although quality and management are improving every year, complications may still arise. Early complications occur within three months of transplant and include bleeding, acute rejection, hemothorax, pneumothorax, and air leaks. Those that occur within one year are considered immediate and include acute rejection, infections, pulmonary thromboembolism, metabolic conditions, and bronchial stenosis or dehiscence. Late complications occur more than a year after transplant and include chronic rejection and post-transplant lymphoproliferative disease [1]. Great care is taken to prevent these complications.

Current management after lung transplant primarily involves lifelong immunosuppressants to prevent rejection, extensive follow-up to monitor possible complications, pulmonary rehabilitation, and overall health management and counseling. While these address many of the major complications discussed, another complication is chronic pain. This is often under-addressed, but is very common and can substantially reduce a patient’s quality of life [4]. Studies have shown that chronic postsurgical pain, defined as surgical field pain for at least three months duration with no other possible causes, can be present in 3% to 85% of patients, with 2% to 25% suffering from a severe form [5]. Pain after lung transplant can be acute, but can also become chronic, persisting for years, which is associated with increased healthcare costs and risk of chronic opioid use [6]. Chronic postoperative pain is poorly understood by all, with no standard form of management. Some patients benefit from the use of medications, others benefit from physical therapy, and many utilize a combination of both, but there is still room for additional measures.

Osteopathic manipulative treatment (OMT) is a hands-on approach to manipulating body structures, resulting in improved systemic homeostasis and patient well-being. The practice incorporates various techniques that utilize the body’s innate ability to self-heal [7]. One of which is the muscle energy technique, where the patient actively contracts their muscles while the physician provides a counterforce resistance. This results in the activation of the Golgi tendon fibers, causing a reflexive inhibition and relaxation of the muscle through the Ia fibers [8]. Balanced ligamentous tension (BLT) is another technique that revolves around the principle that the ligaments of the body provide proprioceptive feedback when tension is balanced along them. After establishing a new balance point to the area being treated by altering the tension of the ligament, it will release, establishing equilibrium at the site of dysfunction [9]. One final technique known as myofascial release (MFR) involves providing a low-amplitude stretch to the myofascial complex of the dysfunctional region for a longer duration of time. The goal is to restore normal length, decrease pain, and improve overall function [10]. The combination of these various techniques can provide value in the management of chronic postoperative pain.

OMT has been widely used as a complementary treatment modality for patients with chronic pain. While literature is limited, few studies have shown the ability of OMT to reduce pain, medication usage, and improve recovery in postoperative patients [11]. OMT addresses somatic dysfunctions, decreases stiffness, improves respiratory function, lymphatic flow, and even immunologic defense [12]. In a literature review of over 240 articles, Randall et. al concluded that OMT in postoperative patients led to significant pain relief, improved bowel function, and decreased length of hospital stay, all while minimizing the use of analgesics and improving comfort [11]. While the literature review incorporated many different surgeries patients were recovering from, lung transplants were not included.

We present a unique case of a 56-year-old female with a past medical history of dermatomyositis status post double lung transplant in 2022, who benefited from the incorporation of OMT to address her chronic pain and stiffness from her procedure.

## Case presentation

A 56-year-old female with a past medical history of dermatomyositis status post bilateral lung transplant in 2022 presented to the clinic with complaints of chronic back pain, diffuse muscle aches, and generalized weakness. The patient described having achy pain and stiffness across her upper and mid-thoracic spine, with the right side being worse than the left, along with tenderness around the surgical scars since the time of surgery. She also reported a history of shingles, which she describes as the possible cause of numbness and tingling across her back. Her medication regimen for immunosuppression and prevention of infection included prednisolone 5 mg daily, mycophenolic acid 360 mg twice a day, tacrolimus 4 mg daily, Zithromax 250 mg three times per week, and Bactrim 400-80 mg once daily. Regarding her chronic pain, she was taking Tylenol and using lidocaine patches as needed, with minimal relief.

On initial presentation, the patient’s vitals were stable, with a temperature of 97.6°F, heart rate of 73 beats per minute, respiratory rate of 16 breaths per minute, blood pressure of 110/76 mmHg, pulse oximeter level of 100%, and BMI of 23.03. The patient’s overall physical exam was benign. Her lungs were clear to auscultation bilaterally, cardiovascular exam had a positive S1 and S2 with a regular rate and rhythm, and no murmurs, rubs, or gallops were noted. Cranial nerves two through twelve were grossly intact with no gross focal defects, and muscle strength was 5/5 with normal range of motion in all upper and lower extremities.

Osteopathic structural exam was significant for bilateral suboccipital muscle hypertonicity, right pectoralis minor strain, right subscapularis hypertonicity, bilateral hemidiaphragm restriction, right latissimus dorsi, serratus anterior, and psoas hypertonicity, and restrictions of the right intercostal muscles of ribs two, three, five, and six. Somatic dysfunctions of T7 extended, rotated, and sidebent right, and L2 extended, rotated, and sidebent right were also noted. The decision was made that OMT would be beneficial for addressing her presenting complaints as well as the structural findings of somatic dysfunctions, hypertonicity, and restrictions. After obtaining consent, a plan to treat the patient with OMT biweekly for the next five months was made. Each session consisted of various techniques, including balanced ligamentous tension (BLT), muscle energy, counterstrain, Still’s technique, myofascial release (MFR), facilitated positional release (FPR), and lumbosacral decompression. The patient was also counseled on strengthening postural muscles at home. All visits utilized similar treatment techniques after a full reassessment of osteopathic structural findings and overall well-being. In addition to OMT, the patient was taking part in a few additional treatment modalities. She had taken part in cardiopulmonary rehab a few times weekly for two months, about four weeks after beginning OMT. She was also doing at-home upper back muscle strengthening and physical therapy prior to her initial OMT visit. Lastly, three months prior to presenting to our clinic, the patient was receiving trigger point injections once every three months. The patient was compliant for the initial four OMT sessions; however, she was then unable to follow up for two months due to scheduling difficulties.

After the initial treatment visit, the patient had improved rib cage excursion as felt by palpation in comparison to her pre-treatment assessment. The patient’s pain was assessed utilizing a pain scale from one to 10, with 10 being the worst pain she had ever felt. She reported a baseline pain rating of six daily. After the initial visit, she reported an immediate reduction in pain from four to three. Subsequent visits further extended the pain relief from multiple days to now weeks, and the patient reported decreased discomfort when taking deep breaths and decreased tenderness to palpation. Both of which provided severe pain and discomfort before treatment with OMT. At each visit, myofascial release techniques were used to address the adhesions caused by her surgical scars, which helped decrease tenderness around the area. Muscle energy techniques and BLT were used to address the patient’s rib cage restrictions and muscle spasms, resulting in further improvement in rib cage expansion, with some persistent shortness of breath. The patient began to notice her ability to sleep comfortably on her back and her side, as well as pain relief that was now lasting weeks. In her final few visits, the patient reported a consistent pain scale rating of two out of 10 throughout her day, as opposed to her initial visit rating of six. While the patient had a period where she was unable to follow up, she continued to report significant improvement in her back pain with no radiating symptoms, an overall increase in her back strength, and reduction of upper and mid-thoracic stiffness.

## Discussion

Lung transplants save thousands of patients’ lives each year. They give patients suffering from end-stage pulmonary disease an improvement in quality of life that they may have never expected. Although lung transplantation has made significant advancements over the years, postoperative complications still arise. Much of the emphasis on postoperative management of these patients is to prevent and assess for major complications, including infections, bleeding, pulmonary thromboembolism, hemothorax, and much more. While those complications are of utmost importance, an often misaddressed complication is chronic pain postoperatively. Even with good surgical technique and pain control in the perioperative period, postoperative pain may still persist. Initial management of choice often revolves around the use of nonsteroidal anti-inflammatory drugs (NSAIDs) or opioids to deal with pain severity, as well as the potential for calcium channel antagonists and antidepressants for neuropathic pain [13]. Medical management is very beneficial and may help the symptomology greatly, but additional treatment modalities may assist in better controlling the pain and regaining quality of life. OMT is one of those modalities that literature has begun to introduce and show support for its effect in improving postoperative pain.

Our patient’s complaints of chronic back pain, stiffness, weakness, and diffuse muscle aches were addressed utilizing multiple osteopathic techniques. Muscle energy technique and BLT improved the various hypertonic muscles our patient was suffering from while also improving rib cage excursion and thoracic and lumbar somatic dysfunctions. MFR was incorporated to address the restrictions and adhesions at the patient’s surgical site from scarring. After performing the technique, our patient reported a notable decrease in tenderness upon inspiration and palpation of the area. The incorporation of various techniques, including muscle energy, BLT, and MFR, resulted in major improvements that ranged from a decrease in pain lasting weeks to decreased stiffness and an overall improvement in quality of life.

Current literature regarding the use of OMT in postoperative patients is limited, and its incorporation in post-lung transplant patients has not been reported. However, the use of OMT in other surgical specialties has been associated with positive outcomes [11]. In the scope of general surgery, nine studies were found that all resulted in less need for pain medication, less pain, and improved recovery [11]. Similar to our study, the most utilized and effective techniques were MFR, muscle energy, balanced ligamentous tension, as well as cranial manipulation. Six studies have been published demonstrating the benefits of OMT postoperatively in cardiothoracic patients. One study, although it did not show a change in pulmonary function, did conclude a decreased pain intensity at 12- and 52-week follow-ups post coronary artery bypass grafting (CABG) [14]. Another, also focused on post-CABG patients, found beneficial hemodynamic changes in cardiac function, alongside decreased length of stay due to the use of OMT [15]. While improvements in various patient outcomes have been reported as well as seen in our patient, one commonality is the impact OMT has had on pain relief. Further implementation and studies need to be done, focused primarily on the postoperative pain experienced by patients, to solidify the role of OMT in its relief.

Major surgeries such as lung transplants can be imperative for improving a patient’s condition. Treatment, however, continues well past the operative time to ensure an improved quality of life. Chronic postoperative pain is a clinical problem that can worsen quality of life and be detrimental to the patient’s healing process. Current management incorporates the use of close follow-up, various medications for immunosuppression and infection prevention, trigger point injections, and potentially physical therapy. As seen with our patient’s synergistic treatment regimen, OMT can be utilized alongside conventional measures to provide an additional benefit with pain reduction and improvement of quality of life.

## Conclusions

Undergoing major surgery, such as a lung transplant, can provide immense benefit to a patient suffering from end-stage disease. However, with such procedures, there is a potential for chronic postoperative pain. While its current management often includes many forms of medication as well as physical therapy, OMT may further improve the patient’s pain. OMT has been well established in its ability to improve muscular tension, range of motion, as well as somatic dysfunctions. Thus, the various symptoms that arise in the postoperative period involving chronic pain can be addressed utilizing OMT.

This case shows the efficacy of OMT in its ability to aid in the management of chronic postoperative pain after a bilateral lung transplant. The patient’s hypertonic muscles, stiffness, tenderness, and decreased range of motion were all addressed with various techniques in addition to her current medical management, resulting in major improvement in quality of life. This highlights the potential role of OMT in managing postoperative pain and underscores the need for further research to better define its place in standard postoperative care.
